# Community-associated methicillin-resistant *staphylococcus aureus* epidemic clone USA100; more than a nosocomial pathogen

**DOI:** 10.1186/2193-1801-2-133

**Published:** 2013-03-26

**Authors:** Jill C Roberts

**Affiliations:** Department of Environmental and Occupational Health, University of South Florida, College of Public Health, Tampa, FL 33612 USA

**Keywords:** *Staphylococcus*, PFGE, Spa, MRSA, USA100

## Abstract

**Background:**

We characterized 100 USA100 epidemic MRSA from individuals in the community with no known healthcare-associated risk factors.

**Findings:**

Molecular epidemiology demonstrated 21 pulsed-field types and six *spa* types. SCC*mec* typing demonstrated that all of the strains possess the type II cassette. The staphylococcal enterotoxin D virulence gene was also present.

**Conclusion:**

Characterization of USA100 MRSA in the community illustrated the importance of nasal carriage, and the genetic diversity of the USA100 clone.

Remarkably in a mere 40 year evolutionary time period, *Staphylococcus aureus* has produced multiple highly successful antibiotic-resistant epidemic clones. Among the methicillin-resistant *S. aureus* (MRSA), are the extensively researched USA300 and the less well characterized USA100 MRSA. Historically, USA300 was described as a community-associated MRSA (CA-MRSA) causing disease in healthy individuals (Chambers 2005; McDougal et al. 2003). Recently the USA300 CA-MRSA designation has been challenged as this pathogen is repeatedly identified as the cause of hospital-acquired MRSA (HA-MRSA) infection. Regardless of the environment from which the strains are isolated, the complement of virulence factors demonstrated in USA300 strains is identical. In contrast, USA100 is considered an HA-MRSA and a cause of invasive infection primarily among persons with healthcare-associated risk factors (Klevens et al. 2007; Limbago et al. 2009). Although detected in nasal swabs from non-institutionalized individuals (Tenover et al. 2008), USA100 MRSA have not been well described in the community environment, the goal of the present study. Herein we characterize 100 USA100 MRSA identified within a collection of isolates from individuals with no known healthcare contact by pulsed-field type (PFT), *spa* type (ST), SCC*mec* type, and PCR identification of virulence factors. Our results are compared to those reported for USA100 healthcare-associated isolates.

A total of 100 CA-MRSA from a previously described report were used in this study (Roberts et al. 2006). The control, USA100 (NRS382), was obtained from the Network on Antimicrobial Resistance in *S. aureus* (NARSA).

PFGE was performed as previously described (Roberts et al. 2006). Genomic DNA from 100 isolates was extracted using the MagNA Pure^®^ and the MagNA Pure LC DNA Isolation Kit III (Roche Diagnostics, Indianapolis, IN) according to manufacturer’s instructions. Typing using the polymorphic region of the *spa* gene and SCC*mec* characterization were performed as previously described (Oliveira & de Lencastre 2002; Shopsin et al. 1999). PCR detection of *lukPV* (Lina et al. 1999), *seb*, *tst*, *sed* (Monday & Bohach 1999) and psm*α* (Wang et al. 2007) genes was performed as previously reported.

Following our report of USA300 among community-associated *S. aureus* (Roberts et al. 2006) our laboratory continued to characterize MRSA from individuals with no known healthcare-associated risks using PFGE. Although the majority of isolates added to our collection are USA300 MRSA, we have also identified a large number of USA100 MRSA from the same population. Previous studies which have characterized USA100 strains primarily from healthcare environments by PFGE have demonstrated great diversity in the USA100 pulsed-field types (PFTs) (McDougal et al. 2003; Limbago et al. 2009; Tenover et al. 2008). Similarly, our study of community-associated USA100 MRSA using PFGE illustrated substantial diversity among the strain patterns as represented by 21 PFTs for the 100 isolates (Simpson’s discriminatory index = 0.920). Reportedly, USA100 MRSA preferentially classify in three PFGE patterns (40.7% of strains) among mostly hospital-associated isolates (Limbago et al. 2009). In the present study, the majority of community USA100 strains possess two PFTs (44% of strains) (Figure 1).

**Figure 1:**
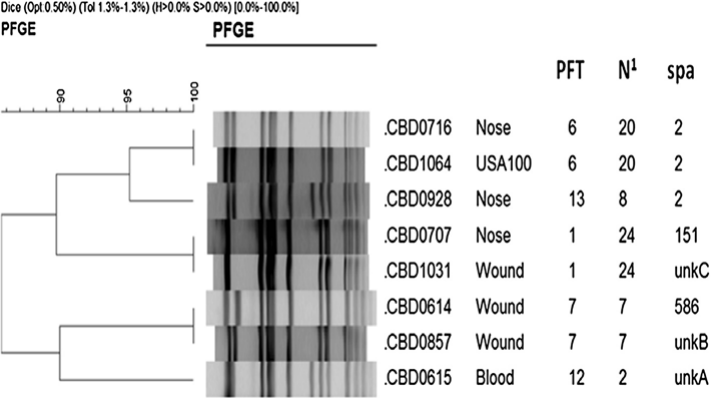
**Variation in pulsed-field Patterns of USA100 MRSA.** N^1^ Number of isolates included in the PFT (pulsed-field type) including control strains. Figure 1 Pulsed-field gel electrophoresis classified the majority of the USA100 strains to PFTs 1 (24 isolates including the control strain USA100/NRS382) and PFT 6 (20 isolates). Representative strains for each of the six *spa* types are shown included three isolates which possessed known repeats but not known repeat patterns and therefore could not be typed (unknown unkA-C). The two typing techniques were not always in agreement as demonstrated by isolates CBD0707 and CBD1031 which possess the same pulsed-field type (Chambers 2005) but different *spa* types (151 and unkC).

Nasal colonization studies have identified USA100 MRSA as the most common MRSA colonizing non-institutionalized individuals in the United States (Tenover et al. 2008). Characterization of these isolates is important in part because of the established link between *S. aureus* nasal carriage and staphylococcal disease. Tenover et al. specifically queried nasal strains and found 44.8% of isolates possess USA100 PFTs (Tenover et al. 2008). In the present study we worked in the opposite direction querying first for USA100 PFTs then identifying etiology. Given the results of the aforementioned study, it is consistent that we found 47 isolates (47%) of our strains identified as USA100 were from nasal swabs (Table 1). Other common sources of USA100 MRSA were wound (27%) and blood samples (13%) consistent with the prevalence of USA100 MRSA in invasive infections (Klevens et al. 2007; Limbago et al. 2009).

**Table 1:** **Summary of results for 100 USA100 Epidemic MRSA**

	Molecular typing			Virulence factor PCR	
	USA100	SCC***mec*** II	***spa*** type^1^	***sed***	***psmα***
Nose	47	47	2,151	45 (96%)	47 (100%)
Wound	27	27	2,586,C	26 (96%)	27 (100%)
Blood	13	13	2,A	12 (82%)	13 (100%)
Urine	4	4	2	3 (75%)	4 (100%)
Other	4	4	2,151,B	3 (75%)	4 (100%)
Lung	3	3	2	3 (100%)	3 (100%)
Unknown	2	2	2	2 (100%)	2 (100%)
Total	100 (100%)	100 (100%)	NA	94 (94%)	100 (100%)

^1^*spa* types identified for each disease etiology are listed. A-C are unknown *spa* types.

Previous studies have demonstrated USA100 MRSA are relatively uniform in SCC*mec* type and *spa* genes, typically containing SCC*mec* II and *spa* type 2 (TJMBMDMGMK) (McDougal et al. 2003; Limbago et al. 2009; Tenover et al. 2008). In rare cases, USA100 isolates have been identified which contain SCC*mec* IV (Tenover et al. 2008). We performed SCC*mec* typing and determined that all of our community USA100 harbor SCC*mec* type II (Table 1). We then performed *spa* typing on every other isolate (a total of 50 isolates) identifying six *spa* types (Table 1). To our knowledge, McDougal et al. (McDougal et al. 2003) is the only published study with *spa* typing results for a large number of USA100 isolates and the HA-MRSA reported therein were uniformly *spa* type 2 (McDougal et al. 2003). A total of 90% of our isolates were also *spa* type 2 (Table 1) and the additional *spa* types (Table 1) we identified is consistent with data (unpublished) for USA100 strains in the *spa* database (SpaServer.ridom.de).

Virulence factor characterization for USA100 MRSA has largely centered on the presence of staphylococcal enterotoxin D. The presence of the *sed* gene in a MRSA strain is in fact considered highly predictive of USA100 (Limbago et al. 2009; Tenover et al. 2008). The percentage of *sed* positive strains by PCR in our isolates was higher than previously reported (94%) (Limbago et al. 2009; Tenover et al. 2008) (Table 1). Additional virulence genes were selected based on their ability to cause diseases in the community including toxic shock, food poisoning (*seb*), necrotizing fasciitis and necrotizing pneumonia (*psmα* and *lukPV*). All of our isolates were positive for the *psmα* gene (Table 1), however previous studies have demonstrated that USA100 strains lack PSM protein production despite the presence of the gene (Wang et al. 2007). Finally, although toxic shock toxin, Panton-Valentine leukocidin, and staphylococcal enterotoxin B have rarely been reported in USA100 MRSA (Limbago et al. 2009), we failed to detect any of these virulence factors among our community USA100 MRSA (data not shown).

In conclusion, our study has demonstrated that USA100 MRSA are present in the community among individuals with no known healthcare contact. Comparison to existing studies has illustrated that the pathogen is essentially identical with respect to diversity of pulsed-field types, anatomical location, SCC*mec* type and virulence factor complement irrespective of the environment from which it was isolated. Perhaps the most significant finding of our study is that when USA100 is found in the community it is most often in the nose. It is tempting therefore to hypothesize that the success of this epidemic clone is partially related to its ability to silently colonize a host. Considering USA100 is the leading cause of invasive MRSA infection, that almost all vancomycin-resistant and intermediate *S. aureus* strains are USA100, and that antibiotic resistance in USA100 isolates is increasing, the importance of continued surveillance for this pathogen cannot be understated.

This study was supported by U.S. Army Research, Development and Engineering Command, contract W911SR-07-C-0084. Reference strains were provided by the Network on Antimicrobial Resistance in *Staphylococcus aureus* (NARSA).
